# α-pinene regulates *miR-221* and induces G_2_/M phase cell cycle arrest in human hepatocellular carcinoma cells

**DOI:** 10.1042/BSR20180980

**Published:** 2018-12-11

**Authors:** Qiuxiang Xu, Ming Li, Mengdie Yang, Jiebo Yang, Jingjing Xie, Xinshuo Lu, Fang Wang, Weiqiang Chen

**Affiliations:** 1 School of Basic Medicine, Guangdong Pharmaceutical University, Guangzhou Higher Education Mega Center, Outer Ring East Road No. 280, Panyu District, Guangzhou 510006, Guangdong Province, China; 2 Guangdong Province Precise Medicine and Big Data Engineering Technology Research Center for Traditional Chinese medicine, Guangdong Pharmaceutical University, Guangzhou Higher Education Mega Center, Outer Ring East Road No. 280, Panyu District, Guangzhou 510006, Guangdong Province, China

**Keywords:** α-pinene, anti-hepatoma carcinoma, apoptosis, in vitro, miR-221, miRNA

## Abstract

The naturally occurring compound α-pinene induces cell cycle arrest and antitumor activity. We examined effects of α-pinene on cell cycle regulation in hepatocellular carcinoma cells (HepG2) cells to establish a foundation for its development as a novel treatment for hepatocellular carcinoma (HCC). HepG2 cells treated with α-pinene exhibited dose-dependent growth inhibition as a result of G_2_/M-phase cell cycle arrest. Cell cycle arrest was associated with down-regulated cyclin-dependent kinase 1 (CDK1) and *miR-221* levels and up-regulated levels of CDKN1B/p27, γ-H2AX, phosphorylated ATM, phosphorylated Chk2 and phosphorylated p53. Our observations are consistent with a model in which α-pinene inhibits *miR221* expression, which leads to G_2_/M-phase arrest and activation of CDKN1B/p27-CDK1 and ATM-p53-Chk2 pathways that suppress human hepatoma tumor progression. Additionally, α-pinene was found to trigger oxidative stress and induce apoptosis of HepG2 cells. α-pinene, therefore, represents a potential chemotherapeutic compound for the treatment of HCC.

## Introduction

The incidence of hepatocellular carcinoma (HCC), a highly malignant cancer, is increasing dramatically with annual new cases estimated at 600,000 worldwide [1,2]. Relatively, few cases can be successfully treated by surgery and the 5-year survival rate for HCC is less than 9% [3]. This is a very low treatment efficacy compared with other major malignancies. Therefore, it is an urgent need to identify novel, effective chemotherapeutic drugs to improve HCC patients’ survival and life quality.

**Figure 1:**
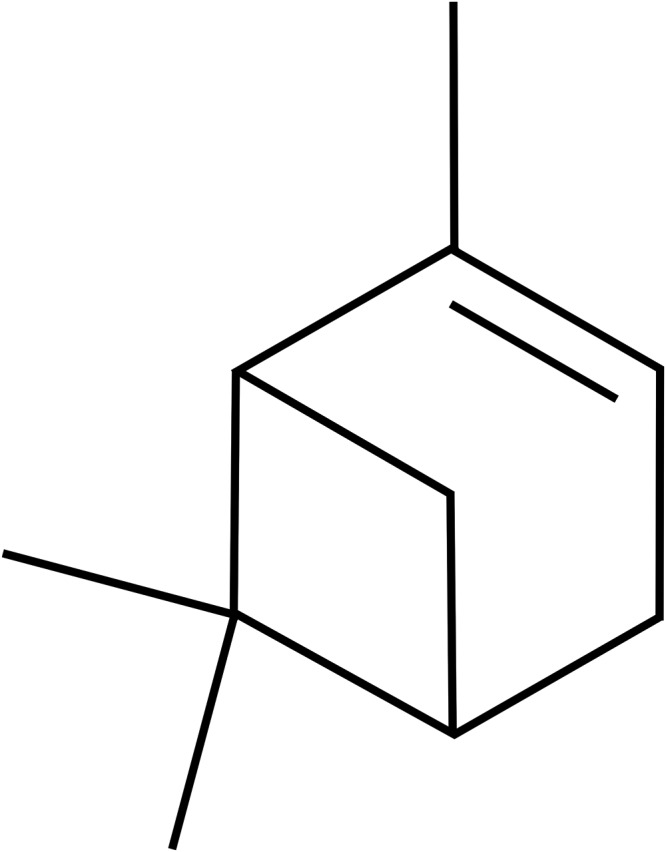
The chemical structure of α-pinene.

The naturally occurring compound α-pinene (Figure 1) can be readily isolated from pine needles and reportedly exhibits antitumor [4,5], antimycotic [6,7] (Jeong, 2007 #13; Pichette, 2006 #12; Pichette, 2006 #12), and antianaphylactic [8] activities. Of particular interest, α-pinene has shown potential as an inhibitor of cell proliferation, inducing cell cycle arrest and suppressing angiogenesis in various cancers through effects on multiple molecular targets and signaling pathways [9].

HepG2 cells are derived from human hepatoblastoma and have high degree of malignancy. And their intrinsic activity of drug-metabolizing enzymes is stable and does not decrease with the increasing passages [10]. We examined the molecular mechanisms underlying α-pinene antihepatoma activity using HepG2 cells, laying a foundation for the development of α-pinene as a novel anticancer drug.

miRNAs are a small, noncoding RNAs that function in post-transcriptional regulation of gene expression. miRNAs are known to regulate the expression of oncogenes and tumor suppressor genes, and they have thus become the focus of research on anticancer drug mechanisms [7,8]. *miR-221* is a key regulatory miRNA [9], the expression of which is increased in HCC, compared with normal hepatic tissue. *miR-221* plays an important role in HCC tumorigenesis, possibly through specific down-regulation of CDKN1B/p27 [11,12]. Indeed, CDKN1B/p27 is a direct target of *miR-221* [13] and when *miR-221* is increased the expression of CDKN1B/p27 is down-regulated [12]. While CDKN1kB/p27 is thought to regulate the G1/S phase transition, research has shown that CDKN1B/p27 can bind to and inhibit the CDK1/cyclin B1 complex to block the cell cycle at G_2_/M phase [11]. Additionally, an active cyclin-CDK protein kinase complex promotes phosphorylation of a variety of proteins involved in cell cycle regulation. Two categories of CDK inhibitors (CDKIs) are recognized: the p16 family including p16, p15, p18, and p19 that specifically inhibit CDK4 and CDK6; and the p21 family including p21, CDKN1B/p27, and p57 that exhibit broad-spectrum CDK inhibition [14]. Thus, inhibition of *miR-221* expression, thereby increasing CDKN1B/p27 activity might effectively inhibit HCC development.

We examined whether α-pinene might act to regulate the expression of *miR-221* and relevant signaling pathways impacting cell cycle dynamics in response to DNA damage involved in HCC development. In response to DNA damage, activated ATM rapidly phosphorylates p53 on Ser15. Phosphorylated p53 dissociates from MDM2 and binds transcription factor CBP/300 which leads to acetylation of the carboxyl-terminal lysine 382 residue of p53 and completion of the damage-repair process [15,16]. ATM also activates Chk2 in response to DNA damage signals following exposure to ionizing radiation or chemotherapeutic agents [17]. We used Western blot analysis, immunofluorescence detection, and qPCR to examine cell cycle-related key regulatory factors (*miR-221*, CDKN1B/p27, cyclin-dependent kinase 1 [CDK1], γ-H2AX, H2AX, phos-ATM [Ser1981], ATM phos-Chk2 [Thr68], Chk2 and phos-p53) in HepG2 cells.

Reactive oxigen species (ROS) not only play an important role in redox regulation during normal physiological functions but are highly reactive molecules that have the potential to cause cellular damage, including damaging DNA, RNA, and proteins and degrading essential cellular molecules [18], which will lead to cells death. The mechanisms of ROS-induced apoptosis typically include receptor activation, caspase activation, Bcl-2 family proteins, and mitochondrial dysfunction [19]. Additionally, ROS-induced apoptosis is associated with decreased generation of glutathione (GSH) levels and the loss of redox homeostasis [20]. N-acety1-L-cysteine (NAC) is an amino with a sulfhydry1 group, it promotes the generation of glutathione, the principle intracellular antioxidant molecule. Thus, it acts as a direct ROS scavenger [21,22]. We performed experiments with DCFH-DA method and Annexin V-FITC/PI to examine whether the increasing of intracellular ROS levels would lead to the apoptosis of HepG2 cells. These studies provide novel molecular insights into the biological basis of potential anticancer efficacy of α-pinene.

## Materials and methods

### Materials

The following reagents were used: α-pinene, resveratrol (RSV), MTT (Sigma–Aldrich, St Louis, MO, U.S.A.); TRIzol Reagent (Life Technologies, Inc., Rockville, MD, U.S.A.); Lipfofectamine^TM^ 3000 Reagent (Invitrogen, CA, U.S.A.); Prime Script^TM^ RT Reagent Kit and SYBR^®^ Premix Ex Taq^TM^II (Takara Bio, Otsu, Japan). *miR-221* and U6 specific primers for reverse transcription and PCR were purchased from Ribobio CO. LTD, Guangzhou, China. CDKN1B/p27 was purchased from Abcam, Cambridge, U.S.A. γ- H2AX, H2AX, phos-ATM (Ser1981), ATM, phos-Chk2 (Thr68), Chk2, p53, and phos-p53 were purchased from Cell Signaling Technology Inc., U.S.A.

### Cell culture

Liver cancer HepG2 cells, breast cancer MCF-7 cells, lung cancer A549 cells, and neuroma cancer PC-12 cells were obtained from the China Center for Type Culture Collection of Wuhan University. Cells were cultured in DMEM containing 10% new-born calf serum, 100 U/ml penicillin and 100 μg/ml streptomycin and incubated at 37°C in a humidified atmosphere containing 5% of CO_2_. Log phase cells were collected after several passages. DMSO concentration was maintained below 0.1%.

### MTT assay

Cells in logarithmic phase were harvested, adjusted to 5 ×10^4^ cells/ml, and seeded into 96-well culture plates at 100 μl per well. At the beginning, cells were exposed to 0, 2, 4, 8, 16, 32, 64, 128, 256 μmol/l or higher concentrations of α-pinene for 24 h. RSV was used as a positive control for anti-HCC activity and added to a concentration of 128 μmol/l [23]. After treatment, 5 mg/ml of MTT was added and cells were incubated at 37°C in the darkness for 24 h. After discarding the supernatant, 75 μl of DMSO was added and plates were placed on a rotary shaker for 15 min. A Bio-Rad iMark microplate reader (Richmond, CA, U.S.A.) was used to determine the absorbance of each well at 570 nm.

### Cell cycle analysis

Flow cytometry (FCM) was used to determine cell cycle distribution. Briefly, after treatment with 0, 16, 32, or 64 μmol/l of α-pinene for 24 h, HepG2 cells were harvested and fixed in 70% ethanol overnight at 4°C. Cells were subsequently resuspended in 0.5 ml and 50 mg/l PI staining solution, kept in the darkness at room temperature for 30 min, and analyzed using a BD Accuri™ C6 Plus System (BD Biosciences, San Jose, U.S.A.). The cell cycle distribution was determined using ModFit LT^TM^ software.

### Quantitative real-time PCR analysis

HepG2 cells cultured in six-well plates were treated with 64 μmol/l α-pinene for 24 h. TRIzol reagent was used to extract total RNA according to standard procedure. Prime Script^TM^ RT Reagent Kit (Takara Bio, Otsu, Japan) with Oligo dT primer or Bulge-Loop^TM^ miRNA qRT-PCR (Ribobio CO. LTD, Guangzhou, China) with Bulge-Loop^TM^ specific primer were used for reverse transcription. Quantitative PCR was performed using aCFX96 real-time PCR Detection System and standard conditions as described for SYBR^®^ Premix Ex Taq^TM^ II (Takara Bio, Otsu, Japan). Experiments were performed in triplicate. Samples were normalized to internal controls and fold changes were calculated based on the formula 2^−ΔΔ*C*^_T_. RNU6B(U6) was used as internal control for miRNA and β-actin as internal control for mRNAs. Primers were based on Genebank sequences and designed using Express Primer 310 software. BLAST was used to confirm homology. CDKN1B/p27, CDK1M, and β-actin primers were synthesized by the LiuheHuada company, Beijing, China. Primer sequences: CDKN1B/p27 (forward, 5′- AAA CTA CAG GTC AAG TGG TAGC CA -3′, reverse, 5′- GTC TGT AGT AGA ACT CGGG -3′), CDK1 (forward, 5′- CCA GGA GTT ACT TCT ATG CCT GA-3′, reverse, 5′- TCC TGC ATA AGC ACA TCC TGA -3′), β-actin (forward, 5′- TCA CCC ACA CTG TGC CCA TCT ACGA-3′, reverse, 5′- CAG CGG AAC CGC TCA TTG CCA ATGG -3′).

### Cell transfection

Lipofectamine^TM^ 3000 was used to introduce *miR-221* mimic into HepG2 cells. HepG2 cells were seeded into six-well plates and 70–90% confluent at the time of transfection. Lipofectamine^TM^ 3000 and *miR-221* mimic were diluted in Opti-MEN^TM^ Medium. Diluted Lipofectamine^TM^ 3000 and *miR-221* mimic were combined at a 1:1 ratio and incubated at room temperature for approximately 15 min. The complex was added to the HepG2 cells for 6–16 h at 37 °C. Subsequently, α-pinene was added to the cells at a concentration of 64 μmol/l and incubated for another 24 h. Cells were then harvested for qPCR analysis.

### Western blot analysis

HepG2 cells treated with 0, 16, 32, or 64 μmol/l of α-pinene for 24 h were harvested by incubating cells in ice-cold cell lysis buffer for Western blot analysis (Beyotime Biotechnology, Shanghai, China). Protein concentration was determined by using a BCA kit (Beyotime Biotechnology, Shanghai, China) according to manufacturer’s instructions. Approximately 20 μg of total protein was separated by 12% SDS/PAGE and transferred to PVDF membrane. The membrane was blocked using 5% (w/v) nonfat milk at room temperature for 1 h, then incubated with primary antibodies (specific for CDKN1B/p27, CDK1, γ- H2AX, H2AX, phos-ATM [Ser1981], ATM, phos-Chk2 [Thr68], Chk2 or phos-p53) in 5% nonfat milk overnight at 4°C. Secondary antibodies conjugated with horseradish peroxidase were diluted 1:2000 in 5% nonfat milk and incubated for 1 h at room temperature. Antibody signals were detected by ECL and analyzed using ImageJ software. β-actin was used as a protein-loading control.

### Immunofluorescence analysis

Cells were fixed in precooled (−20°C) acetone-methanol solution for 15 min. Primary antibodies anti-γH2AX (upstate, 0.5 μg/ml) and antiphospho-ATM (S1981) (cell signaling, 1:100) were incubated at 4°C overnight. After washing, secondary antibodies FITC-Donkey antirabbit IgG (1:200, Jackson Immuno Research Lab) and Rhodamine-Donkey antimouse IgG (1:200, Jackson Immuno Research Lab) were incubated at room temperature for 1 h. The cells were then covered with VECTASHIELD mounting medium containing DAPI (VECTOR Lab Inc., Burlingame, CA94010). Cells were visualized using a fluorescence microscope (Carl Zeiss, Axiovert 200).

### ROS measurement

Cellular ROS was measured with DCFH-DA, which was employed to measure ROS production. HepG2 cells were treated with 0, 16, 32, 64 μmol/l of α-pinene, and 10 mmol/l of NAC+64 μmol/l α-pinene (pretreated with 10 mmol/l NAC for 2 h and then exposed to 64 μmol/l of α-pinene) for 24 h. DCFH-DA was then added to a final concentration of 10 μmol/l and incubated for 20 min at 37°C. Fluorescence intensities were monitored by fluorescence microscopy (Carl Zeiss, Axiovert 200), and the images were semiquantitatively analyzed with ImageJ.

### Apoptosis analysis

FCM was used to analyze early and late phases of apoptotic cells. Briefly, after treatment with 0, 16, 32, 64 μmol/l of α-pinene, and 10 mmol/l NAC+64 μmol/l of α-pinene for 24 h, HepG2 cells were collected and washed twice with cold PBS. After resuspending in Binding Buffer and FITC-Annexin V for 15 min, PI staining solution was added. The cells were kept in the darkness and analyzed using a BD Accuri™ C6 Plus System (BD Biosciences, San Jose, U.S.A.) within 30 min.

### Statistical analysis

All experiments were repeated at least three times. Data were analyzed using SPSS 19.0 software (SPSS 19.0 for Windows; SPSS, Inc. Chicago, IL, U.S.A.) and presented as mean ± S.D. for three separate experiments. Differences amongst three or more groups were analyzed by one-way ANOVA for multiple comparisons. *P*<0.05 was considered significant.

## Results

### α-pinene induced the cancer cells toxicity

**Figure 2:**
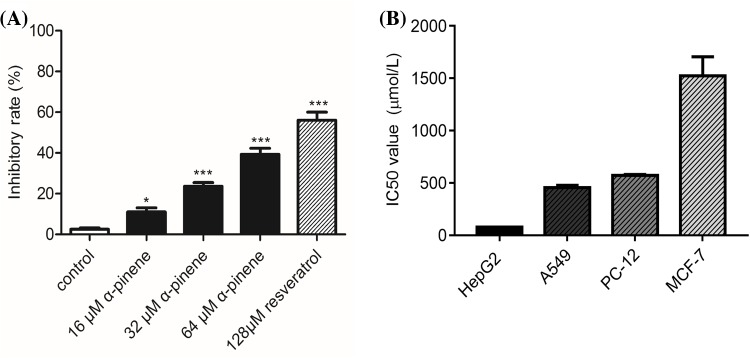
Inhibitory rate and the IC50 value of α-pinene on tumor cells (**A**) Inhibitory effects of α-pinene on HepG2 cell proliferation. MTT assay results. HepG2 cells were treated for 24 h with 0, 16, 32, 64 μmol/l of α-pinene. Positive control cells were treated with 128 μmol/l resveratrol. (**B**) The IC50 value of a-pinene on HepG2 cells, A549 cells, PC-12 cells and MCF-7 cells.

According to the MTT assay results, after exposed to α-pinene for 24 h, as shown in Figure 2A, α-pinene inhibited the proliferation of HepG2 cells in a dose-dependent manner. At a concentration of 64 μmol/l α-pinene inhibited HepG2 cells proliferation by approximately 39.3 ± 4.2%. The proliferation activities of the cells were inhibited in an order from strong to weak, which were HepG2 cells, A549 cells, PC-12 cells, and MCF-7 cells. And the IC50 value of a-pinene on each cell lines was displayed in Figure 2B. So we chose HepG2 cells for the subsequent research.

### α-pinene induced G_2_/M phase cell cycle arrest

**Figure 3:**
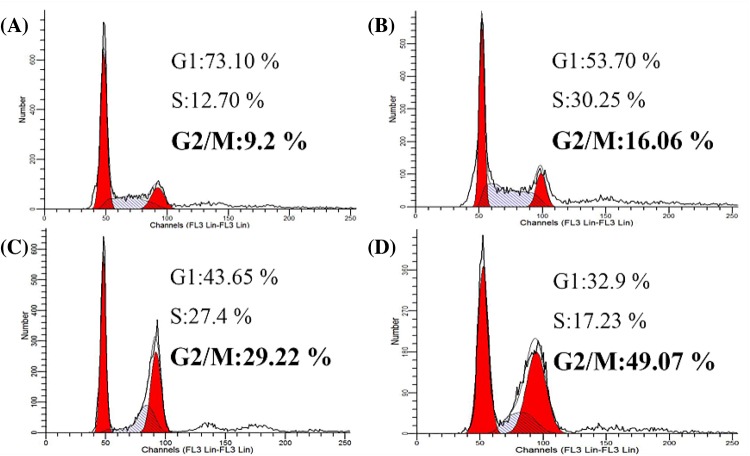
Cell cycle distribution following treatment with α-pinene. (**A**) Nontreated control cells. (**B**) Treatment with 16 μmol/l α-pinene for 24 h. (**C**) Treatment with 32 μmol/l of α-pinene for 24 h. (**D**) Treatment with 64 μmol/l of α-pinene for 24 h.

FCM was used to analyze the cell cycle distribution of HepG2 cells following 24 h of treatment with increasing concentrations of α-pinene. As shown in Figure 3, treatment resulted in arrest at G_2_/M phase, and the percentage of HepG2 cells at G_2_/M was 9.2, 16.06, 29.22, and 49.07% in the 0, 16, 32, and 64 μmol/l α-pinene treatment groups, respectively.

### Effects of α-pinene treatment on *miR-221* and CDKN1B/p27-CDK1 signaling pathway components

**Figure 4:**
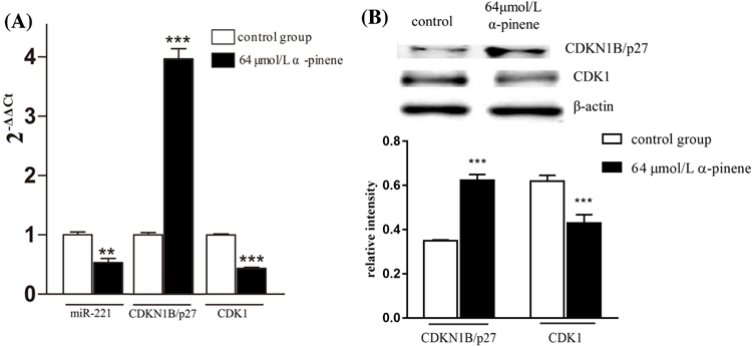
Effects of α-pinene on expression of miR-221, CDKN1B/p27, and CDK1 in HepG2 cells (**A**) qPCR analysis of α-pinene induced changes in *miR-221*, CDKN1B/ p27, and CDK1 expression. (**B**) Western blot analysis of α-pinene induced changes in CDKN1B/p27 and CDK1 protein level. Results represent three independent experiments. **P<0.01 and ***P<0.001 (compared with control).

**Figure 5:**
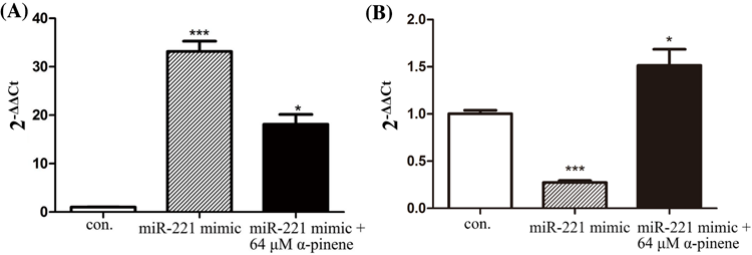
Effects of α-pinene on expression of miR-221 and CDKN1B/p27 in HepG2 cells transfected with miR-221 mimic (**A**) Effects of α-pinene on expression of *miR-221* in HepG2 cells transfected with *miR-221* mimic. (**B**) Effects of α-pinene on expression of CDKN1B/p27 in HepG2 transfected with *miR-221* mimic. Results represent three independent experiments. *P<0.05, and ***P<0.001; n.s: not significant (compared with control).

To better understand the molecular mechanisms by α-pinene induced G_2_/M phase cell cycle arrest in HepG2 cells, we analyzed *miR-221* and CDKN1B/p27-CDK1 signaling pathway dynamics. As shown in Figure 4, α-pinene treatment led to *miR-221* and CDK1 down-regulation, while CDKN1B/p27 was up-regulated. To further study effects of α-pinene on *miR-221* and CDKN1B/p27, we introduced a *miR-221* mimic into HepG2 cells by transfection and treated the cells with 64 μmol/l α-pinene for 24 h. As shown in Figure 5A, in contrast with control cells, *miR-221* in transfected cells was markedly up-regulated. Nevertheless, α-pinene treatment was associated with *miR-221* down-regulation in both control and transfected cells. As shown in Figure 5B, in HepG2 cells transfected with *miR-221* mimic the expression of CDKN1B/p27 was significantly down-regulated. Treatment with α-pinene led to up-regulated CDKN1B/p27 expression.

### Effects of α-pinene treatment on ATM-p53-Chk2 signaling pathway components

**Figure 6:**
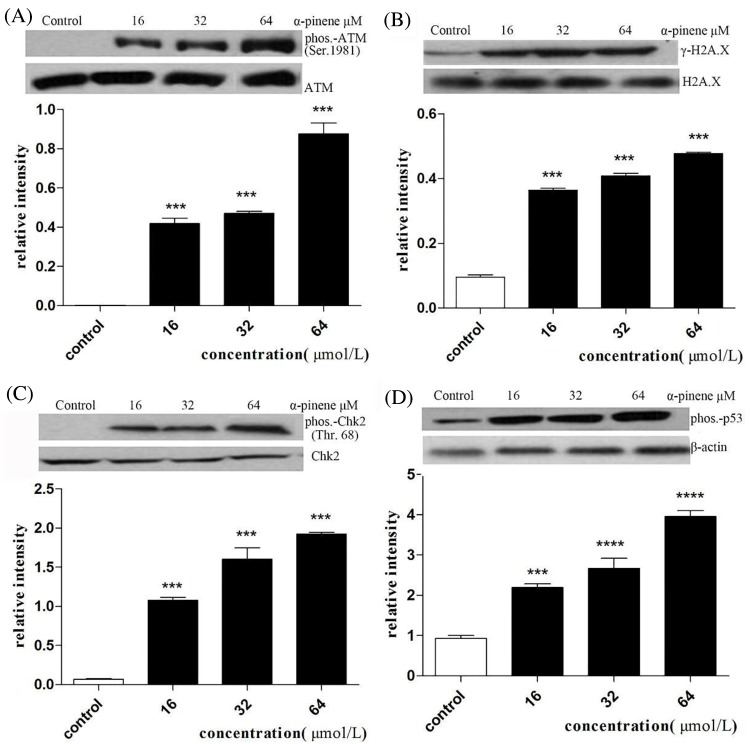
Effects of α-pinene on expression of phosphorylated ATM (Ser1981), γ-H2AX, phosphorylated p53 and phosphorylated Chk2 (Thr68)in HepG2 cells (**A**) relative abundance of γ-H2AX compared with H2AX (**B**) relative abundance of phosphorylated ATM (Ser1981) compared with ATM (**C**) relative abundance of phosphorylated Chk2 (Thr68) compared with Chk2 (**D**) relative abundance of phosphorylated p53 compared with β-actin. Results represent three independent experiments.***P<0.001, ****P<0.0001 (compared with control).

Western blot analysis of proteins involved in cell cycle regulation was performed to better understand the mechanism of α-pinene induced G_2_/M phase arrest of HepG2 cells. As shown in Figure 6, phosphorylated ATM (Ser1981), γ-H2AX, phosphorylated p53 and phosphorylated Chk2 (Thr68) exhibited dose-dependent up-regulation following α-pinene treatment of HepG2 cells.

**Figure 7:**
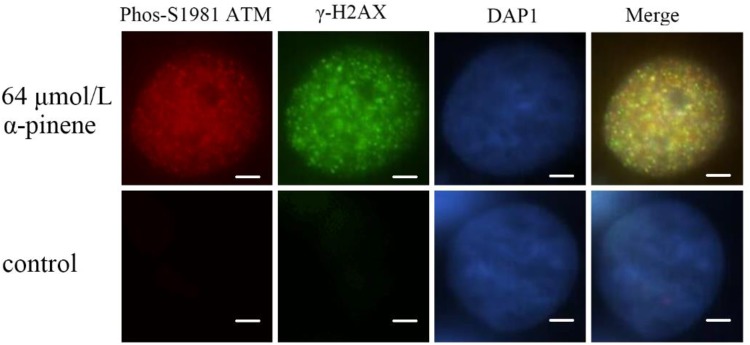
α-pinene treatment led to increased ATM (S1981) phosphorylation and γ-H2AX nuclear foci. HepG2 cells were treated with DMSO (control) or 64 μmol/l of α-pinene for 24 h, followed by double immunofluorescence staining for ATM (S1981) phosphorylation (red), and γ-H2AX (green). Nuclei were counterstained with DAPI (blue). Scale bars represent 5 μm.

Immunofluorescence analysis was used to further clarify effects of α-pinene treatment on ATM and H2AX proteins in HepG2 cells. As can be seen in Figure 7, the nuclear localization of phosphorylated-ATM (S1981) and γ-H2AX in α-pinene treated HepG2 cells was much more pronounced than in nontreated control cells.

### α-pinene triggered oxidative stress and induced apoptosis

**Figure 8:**
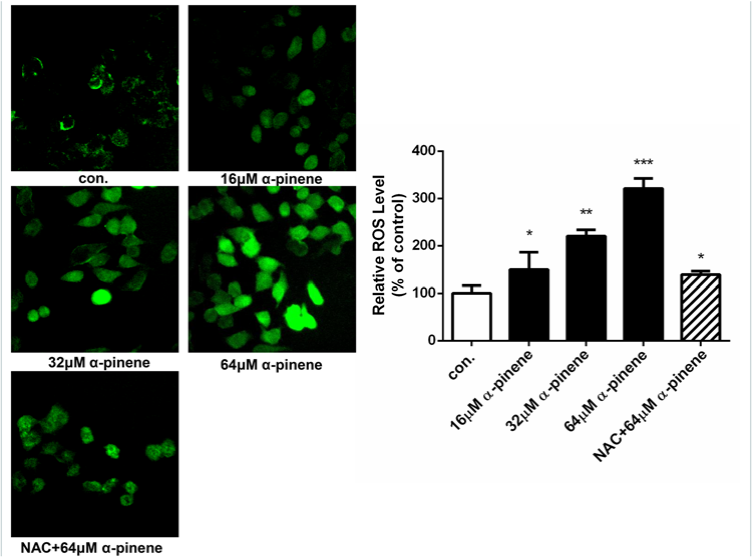
HepG2 cells were exposed to 0, 16, 32, 64 μmol/l α-pinene, and NAC+64 μmol/l α-pinene for 24 h. α-pinene induced ROS level increased and pretreated with NAC decreased the ROS level. Results represent three independent experiments. *P<0.05, **P<0.01, and ***P<0.001, n.s: not significant (compared with control).

**Figure 9:**
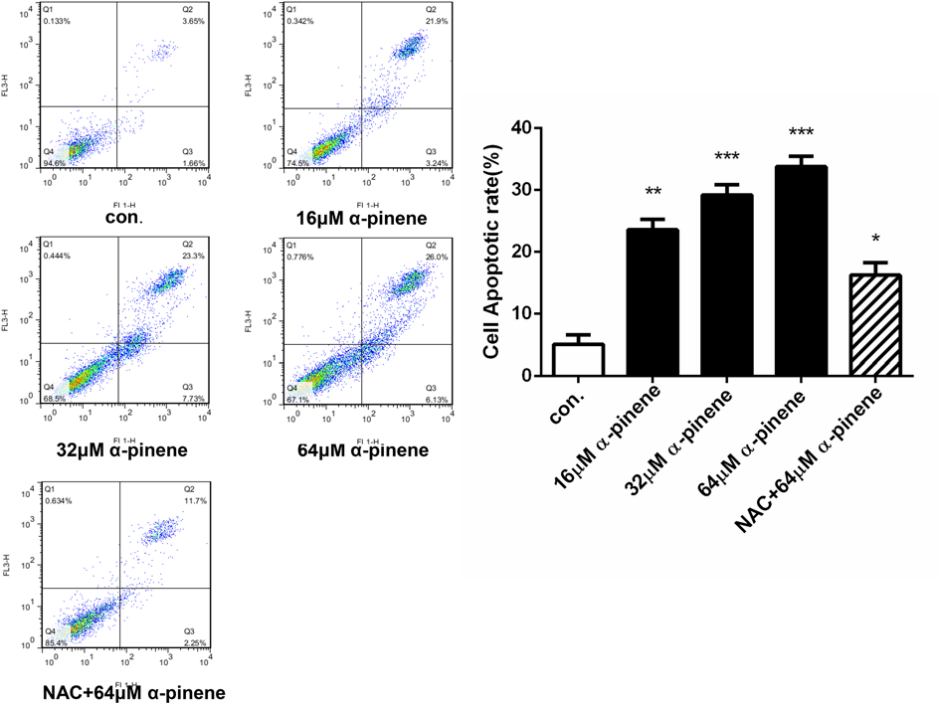
HepG2 cells were exposed to 0, 16, 32, 64 μmol/l α-pinene, and NAC+64 μmol/l α-pinene for 24 h. α-pinene elicited apoptosis in HepG2 cells and pretreated with NAC had protective effective. Results represent three independent experiments. *P<0.05, **P<0.01, and ***P<0.001; n.s: not significant (compared with control).

The cytoyoxic effects of α-pinene on HepG2 cells are probably mediated through ROS production, which is an important intracellular signal of cell proliferation, cell death, and homeostasis, etc. As shown in Figure 8, the brightness of fluorescence and ROS level increased in a concentration-dependent manner, and the differences were significant. When the cells were protected by NAC, the brightness and ROS level decreased clearly. From Figure 9, after treated with various concentrations (0, 16, 32, and 64 μmol/l) of α-pinene, the HepG2 cells apoptotic rate increased significantly, and in comparison with the 64 μmol/l α-pinene treatment group, the apoptotic rate of NAC protective group decreased markedly.

## Discussion

HCC, the predominant primary liver cancer, is the second most common cause of cancer death around the world [24]. However, the conventional therapies perform great side effect on human body. Therefore, it is imperative to find effectively drugs with fewer side effect for the malignant tumor. In this work, α-pinene displayed a promising *miR-221* regulation, cell cycle arrest, ROS production increasing, and proapoptosis effect on HepG2 cells, suggesting that α-pinene impacts cell cycle dynamics in response to DNA damage and induces cytotoxic effect involved in HCC development. The results of the present study may provide a new option for the treatment of HCC.

In the present study, α-pinene was shown to induce G_2_/M phase cell cycle arrest in HepG2 cells, consistent with previous studies using a different HCC cell line, Bel-7402 [5]. However, previouse study showed that RSV arrested GepG2 cells in S phase [25], they showed different mechanism on the growth inhibition of GepG2 cells, so we did not used it as a positive control in the subsequent experiments. The G_2_/M checkpoint is the final opportunity for the repairment of damaged DNA prior to mitosis. To further explore the mechanism of α-pinene effects on HepG2 cells, we analyzed changes induced by α-pinene treatment on *miR-221*, CDKN1B/p27, CDK1, γ-H2AX, H2AX, phosphorylated ATM (Ser1981), ATM, phosphorylated Chk2 (Thr68), Chk2, and phosphorylated p53, all of which have roles in G_2_/M phase cell cycle regulation.

We observed α-pinene treatment-induced down-regulation of *miR-221* and CDK1 and up-regulated expression of CDKN1B/p27. Transfection of HepG2 cells with a *miR-221* mimic successfully increased *miR-221* levels and led to decreased CDKN1B/p27. However, transfected cells treated with 64 μmol/l of α-pinene exhibited down-regulated *miR-221* levels and up-regulation of CDKN1B/p27. This may indicate that α-pinene activates the CDKN1B/p27-CDK1 signaling pathway by the inhibition of pathway inhibitor *miR-221*, leading to G_2_/M-phase cell cycle arrest. Our previous studies showed that α-pinene reduces the expression of cyclinB1 and CDK1 proteins in tumor tissue [4,5]. To determine whether this is related to the α-pinene induced *miR-221*-mediated effects on CDKN1B/p27-CDK1 pathway, activation observed in the current study will require further analysis.

We also found evidence that α-pinene treatment induced dose-dependent up-regulation of γ-H2AX, phosphorylated ATM (S1981), phosphorylated CHK2 (T68), and phosphorylatedp53. These observations suggest that α-pinene can activate the ATM-p53-Chk2 signaling pathway to induce G_2_/M phase arrest, thereby inhibiting HepG2 cell proliferation.

Elevated levels of ROS are thought to be oncogenic, causing damage to DNA, proteins and lipids, promoting genetic instability, and tumorigenesis [26]. α-pinene increased ROS production in a dose-dependent manner. When pretreated with NAC, the ROS production increased less than only treated with 64 μmol/l of α-pinene. Apoptosis is a programmed cell death mediated through the death receptor-mediated extrinsic pathway and the mitochondrial-mediated intrinsic pathway. Our study revealed that α-pinene induced the HepG2 cells apoptosis in a dose-dependent manner, and NAC has a protective effect. These results indicated that the mechanism of α-pinene induced apoptosis of HepG2 cells may be related to oxidative stress.

The current study provides promising *in vitro* evidence that α-pinene effectively modulates *miR-221* levels and activates the CDKN1B/p27-CDK1 and ATM-p53-Chk2 pathways, leading to G_2_/M phase cell cycle arrest of HepG2 cells. In addition, α-pinene increases ROS production and induces apoptosis in GepG2 cells. The pharmacokinetics of α-pinene in liver remains unclear so far, much more work employing additional cellular, and *in vivo* animal model studies will be needed to verify the translational potential of α-pinene as a chemotherapeutic agent useful for the treatment of HCC.

## Abbreviations

ATM: Ataxia Telangiectasia Mutated
CDK1: cyclin-dependent kinase 1
CDKI: CDK inhibitor
DCFH-DA: 2′,7′-Dichlorodihydrofluorescein diacetate
DMSO: Dimethyl sulfoxide
GSH: generation of glutathione
HCC: hepatocellular carcinoma
HepG2: hepatocellular carcinoma cell
NAC: N-acety1-L-cysteine
PI: Propidium Iodide
ROS: reactive oxigen species
RSV: resveratrol
